# Comparing the effect of STan (cardiotocographic electronic fetal monitoring (CTG) plus analysis of the ST segment of the fetal electrocardiogram) with CTG alone on emergency caesarean section rates: study protocol for the STan Australian Randomised controlled Trial (START)

**DOI:** 10.1186/s13063-019-3640-9

**Published:** 2019-08-29

**Authors:** D. Turnbull, A. Salter, B. Simpson, B. W. Mol, E. Chandraharan, A. McPhee, I. Symonds, M. Benton, S. Kuah, G. Matthews, K. Howard, C. Wilkinson

**Affiliations:** 1 School of Psychology, University of Adelaide, Adelaide, South Australia Australia; 2 School of Public Health, University of Adelaide, Adelaide, South Australia Australia; 3 Women’s and Children’s Hospital, Adelaide, South Australia Australia; 4 Department of Obstetrics and Gynaecology, Monash University, Melbourne, Victoria Australia; 5 NHS Foundation Trust, St George’s University Hospitals, London, UK; 6 Adelaide Medical School, University of Adelaide, Adelaide, South Australia Australia; 7 School of Public Health, The University of Sydney, Sydney, New South Wales Australia

**Keywords:** Continuous electronic fetal monitoring, Cardiotocography, ST analysis, Caesarean section, Randomised controlled trial, START

## Abstract

**Background:**

Cardiotocography is almost ubiquitous in its use in intrapartum care. Although it has been demonstrated that there is some benefit from continuous intrapartum fetal monitoring using cardiotocography, there is also an increased risk of caesarean section which is accompanied by short-term and long-term risks to the mother and child. There is considerable potential to reduce unnecessary operative delivery with up to a 60% false positive diagnosis of fetal distress using cardiotocography alone. ST analysis of the fetal electrocardiogram is a promising adjunct to cardiotocography alone, and permits detection of metabolic acidosis of the fetus, potentially reducing false positive diagnosis of fetal distress.

**Methods:**

This study will be a single-centre, parallel-group, randomised controlled trial, conducted over 3 years. The primary hypothesis will be that the proportion of women with an emergency caesarean section on ST analysis will not equal that for women on cardiotocography monitoring alone. Participants will be recruited at the Women’s and Children’s Hospital, a high-risk specialty facility with approximately 5000 deliveries per annum. A total of 1818 women will be randomised to the treatment or conventional arm with an allocation ratio of 1:1, stratified by parity. The primary outcome is emergency caesarean section (yes/no). Statistical analysis will follow standard methods for randomised trials and will be performed on an intention-to-treat basis. Secondary maternal and neonatal outcomes will also be analysed. Additional study outcomes include psychosocial outcomes, patient preferences and cost-effectiveness.

**Discussion:**

Approximately 20% of Australian babies are delivered by emergency caesarean section. This will be the first Australian trial to examine ST analysis of the fetal electrocardiogram as an adjunct to cardiotocography as a potential method for reducing this proportion. The trial will be among the first to comprehensively examine ST analysis, taking into account the impact on psychosocial well-being as well as cost-effectiveness. This research will provide Australian evidence for clinical practice and guideline development as well as for policy-makers and consumers to make informed, evidence-based choices about care in labour.

**Trial registration:**

ANZCTR, ACTRN1261800006268. Registered on 19 January 2018.

**Electronic supplementary material:**

The online version of this article (10.1186/s13063-019-3640-9) contains supplementary material, which is available to authorized users.

## Background

Use of cardiotocography (CTG) is almost ubiquitous in intrapartum care [1]. In fact, continuous CTG is one of the most common procedures undertaken during labour, with routine data collection and other reports from Australia, the setting for STan Australian Randomised controlled Trial (START), showing that it is applied in 60–70% of all labours [2, 3]. While systematic reviews demonstrate benefit from such monitoring [4], important shortcomings exist, notably in relation to risk of increased rates of caesarean section [4, 5] which in turn can be accompanied by short-term and long-term risks to the mother and child [6]. One-third of Australian women now deliver via caesarean section [7], with emergency caesarean section rates of between 18 and 20% [8]. While CTG is also associated with an increase in instrumental vaginal birth when compared with intermittent auscultation, it shows no improvement in overall perinatal death rates or cerebral palsy rates [4].

A promising adjunct to CTG is electronic fetal monitoring which also incorporates ST analysis (STan) of the fetal electrocardiogram. This approach permits detection of metabolic acidosis of the fetus by identifying changes to the ST segment of the unborn baby’s ECG [9] which are correlated with a simultaneously performed CTG, potentially reducing false positive diagnosis of fetal distress. With up to a 60% false positive diagnosis of fetal distress using CTG alone [10], there is considerable potential to reduce unnecessary operative delivery.

The main potential benefit for STan monitoring lies in the associated reduction in use of scalp pH sampling in low emergency caesarean section environments. While the most recent Cochrane meta-analysis [11] found no significant reduction in caesarean section rate in low caesarean section environments, where arguably fewer unnecessary caesarean sections are performed, a statistically significant reduction in the relative risk of women having scalp pH samples was demonstrated. This finding has subsequently been supported in an individual patient data meta-analysis [12].

This has crucial implications for Australian practice, as the indications for a decision for emergency caesarean section are the same as those for deciding to undertake scalp pH sampling. The specific relevance to our trial is that scalp pH testing is variably used in Australia, as its clinical utility has been seriously questioned [13]. However, it can be reasonably argued that if fetal blood sampling was indicated in a European context, a caesarean section would have been indicated in the Australian context.

There are additional factors which suggest that the European trials, as well as the recently published multicentre US study [14] which also showed no reduction in caesarean section, have limited generalisability to Australian practice. Firstly, the four European trials [15–18] included in the Cochrane review [11], which included women having STan for standard indications, had an overall emergency caesarean section rate of 10.6%, in contrast to the emergency caesarean section rate in Australia of approximately 18–20% [8]. Given that Australia has a higher rate of potentially avoidable caesarean sections, this would enhance the degree to which a reduction in such unnecessary caesarean section is possible. Also, US fetal monitoring practice is fundamentally different to that in Australia. The US study [14] included women at low risk for caesarean section who would not be offered CTG monitoring in Australia, and would have a low background risk of caesarean section regardless of monitoring type.

Further, delivery decisions for observed STan events were set at a lower threshold in the US study relative to the European guidelines and the clinical protocol that we report here [19]. Additionally, the use of STan in the US study did not result in fewer caesarean sections in an environment where the alternative to this intervention at full dilatation (i.e. instrumental vaginal delivery) is much less common. Instrumental vaginal delivery occurred in only about 6% of cases in the US study [14], compared to 14% at the study hospital for the current trial [20].

A ‘culture change’ may also be necessary for effective implementation of STan [21]. A programme of continuing education and feedback, involving all of the labour-ward midwives and medical staff, is a strong feature of institutions where STan has been successfully implemented, such as in Norwegian clinical practice [22]. While an education programme was implemented at the beginning of the US trial [14], STan monitoring was applied to an average of only 1.4 women per week in each of the study hospitals, providing exceptionally limited opportunities for development and maintenance of skills. This scenario is exacerbated in an organisational culture where changeover of doctors and midwives, and hence loss of trained STan expertise due to clinical rotations, would be common.

The most recent meta analyses at the time of this publication [23, 24] showed that the use of STan is associated with no difference in the risk of caesarean section (RR 0.93; 95% CI 0.78–1.12) [23] or operative vaginal deliveries for fetal distress (RR 0.87; 95% CI 0.74–1.03) [23] but did show a 36% reduction in metabolic acidosis (OR 0.64; 95% CI 0.46–0.88) [23]. Thus, the concurrence of a low background emergency caesarean section rate (in the European trials) and the use of fetal monitoring in a low-risk population (in the US trial), along with infrequent use and thus less experience and training of clinical staff, suggests that a reduction in caesarean section may be possible in an Australian setting. In fact, our pilot trial of 162 women indicated a 21.2% emergency caesarean section rate for the CTG group, and a 13.4% emergency caesarean section rate for the STan group [25]. The proposed study presents the ideal opportunity to examine STan in an adequately powered randomised controlled trial, the first of its kind in Australia and the first comprehensive trial worldwide.

## Methods

### Hypotheses

We believe that STan monitoring (cardiotocographic electronic fetal monitoring (CTG) plus analysis of the ST segment of the fetal electrocardiogram) of labouring women will result in a reduction in the proportion of emergency caesarean sections when compared with CTG monitoring alone, from 17% to 12%. The primary hypothesis is that the proportion of emergency caesarean sections for women on STan will not be equal to that for women on CTG monitoring alone. The study will be powered to detect an absolute difference of 5% with a conservative two-sided alternative. Our secondary hypotheses are that: there will be similarity in neonatal and maternal clinical outcomes in both treatment arms; STan monitoring will result in improved psychosocial outcomes; STan monitoring will be cost-effective compared to CTG alone; and STan monitoring will be preferred by women and they will be willing to trade-off the disadvantages, such as reduced mobility and the need for a fetal scalp electrode (FSE), in order to obtain advantages such as reduced chance of caesarean section.

### Trial design

**Fig. 1:**
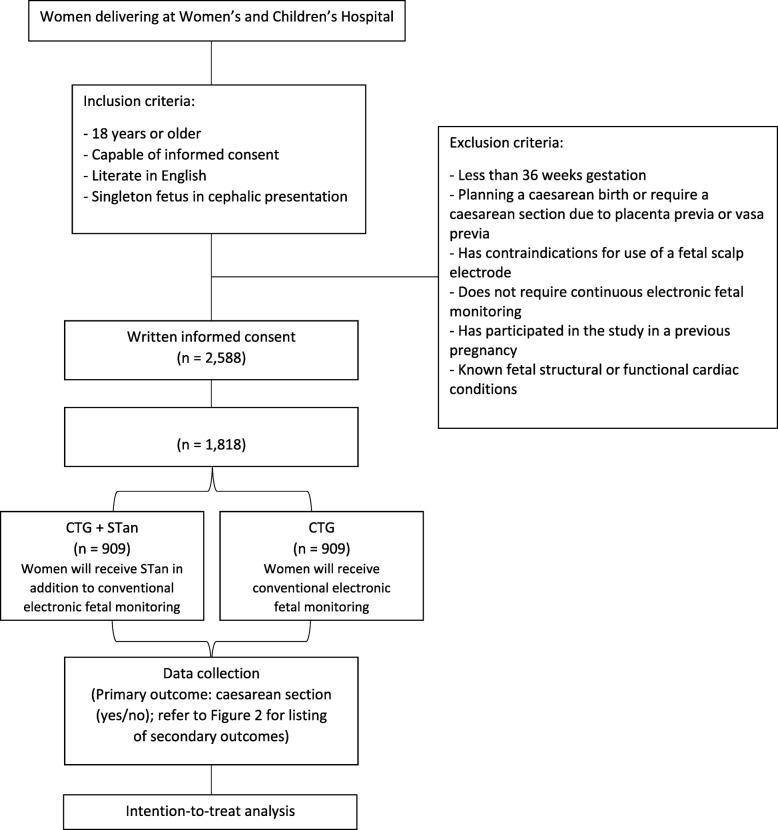
Trial flow chart. CTG cardiotocography, STan ST analysis

**Fig. 2:**
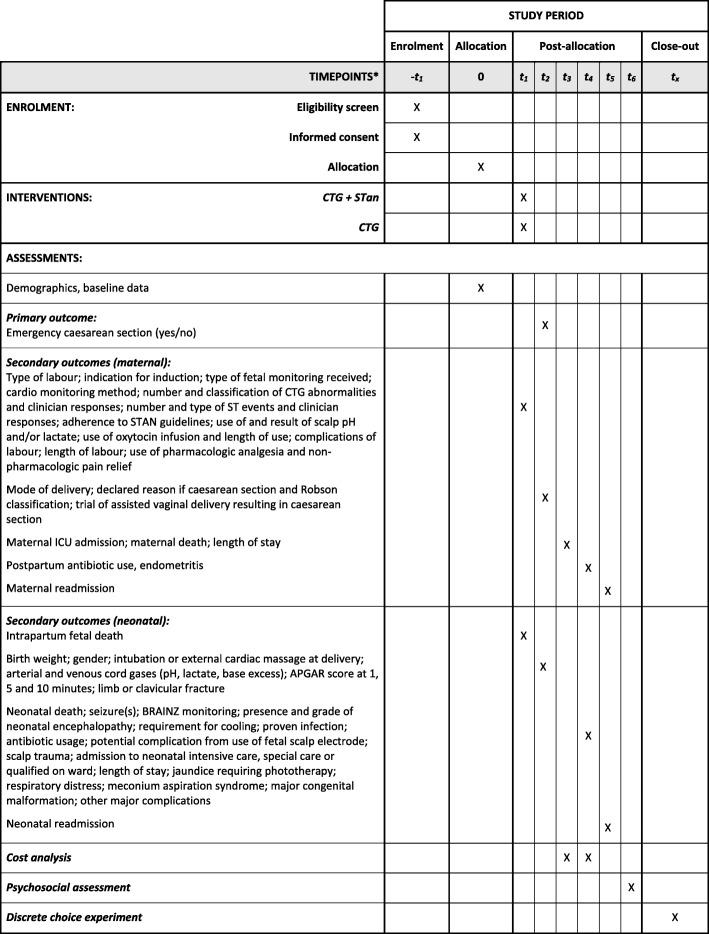
The schedule of enrolment, interventions and assessments. CTG cardiotocography, ICU intensive care unit, STan ST analysis

This study will be a single-centre, parallel-group, randomised controlled trial, conducted over 3 years. Figure 1 provides the study flow and Fig. 2 shows a version of the Standard Protocol Items: Recommendations for Interventional Trials (SPIRIT) figure for the trial. Details of the study methodology are outlined in the SPIRIT checklist in Additional file 1. There will be an intervention group (CTG + STan) and a conventional treatment group (CTG).

### Trial administration, study population and eligibility criteria

The trial is administered by the University of Adelaide, Adelaide, South Australia and the Women’s and Children’s Hospital, Women’s and Children’s Health Network, Adelaide, South Australia. A total of 1818 women who have antepartum or intrapartum risk factors that increase the risk of fetal compromise for which continuous electronic fetal monitoring during labour is recommended will be randomised for the study. Participants will be recruited at the Women’s and Children’s Hospital, a high-risk specialty facility with approximately 5000 deliveries per annum. Women are eligible for the trial if they meet the following inclusion criteria: 18 years or older; capable of informed consent; literate in English; and singleton fetus in cephalic presentation. Women will be excluded from participating if: they are less than 36 weeks gestation; they are planning a caesarean birth or require a caesarean due to placenta previa or vasa previa; they have contraindications for use of a fetal scalp electrode; they do not require continuous electronic fetal monitoring; they have participated in the study in a previous pregnancy; or there are known fetal structural or functional cardiac conditions.

### Sample size

Considering the results of our pilot study [25], current hospital rates for emergency caesarean section and electronic fetal monitoring, and the results of previous research [12], we powered the primary hypothesis on a reduction of emergency caesarean section from 17% to 12%, (i.e. a 5% absolute difference), with 80% power and a conservative two-sided α = 0.05. To detect this absolute difference between the treatment groups, a total sample size of 1634 women is required. After allowing for a very conservative 10% dropout after consent and a further 22% attrition after consent due to no clinical indication for fetal monitoring in labour, and a possible further 5% non-compliance in the STan + CTG arm, consent would need to be gained from at least 2588 women. Given a recruitment period of about 2 years, we estimated that it would be possible to consent 2588 women, of whom 1818 will be subsequently randomised. This sample size would also provide power to detect clinically meaningful differences in the secondary outcomes of interest.

This is achievable at the study hospital. Of 5000 women per year birthing at the Women’s and Children’s Hospital, 12% have elective caesarean sections and do not labour, and approximately 8% are born pre-term, leaving 80% of women to labour at term (4000 women per year). Of these women, approximately 80% have electronic fetal monitoring (3200 women per year). The US study [14] reported a consent and randomisation rate of 25.6%. Assuming a conservative consent and randomisation rate of only 20% of the over 9600 women thus eligible over a 3-year period, 1920 women would be consented and randomised. Given the 1818 consented and randomised women required in our sample size calculation, this sample size should be achievable.

### Ethical aspects and data storage

The randomised controlled trial was approved by the Women’s and Children’s Health Network’s Human Research Ethics Committee (HREC/17/WCHN/14) and is covered by indemnity insurance. Original consent forms will be stored in a locked filing cabinet, in a locked room. Data will be stored on secure servers at the University of Adelaide and the Women’s and Children’s Hospital. Data and consents will be retained for 15 years after the publication of the trial according to the standards required by the Australian Code for the Responsible Conduct of Research [26].

### Study schedule

Women will be approached to ascertain interest in and to seek consent about the study at 32–36 weeks gestation. Consent will be obtained by appropriately trained clinicians and will take place at various locations throughout the hospital including the labour ward, the Women’s Assessment Service, antenatal/gynaecology wards and antenatal outpatient clinics. After ongoing assessment of eligibility during labour, eligible consenting women will be randomised once the decision to electronically monitor the fetus during labour is made. Any excluded or voluntarily withdrawn patients will receive usual care without prejudice. Demographic and clinical baseline data will be collected by the research midwife at or soon after trial entry. Clinical observation will commence at randomisation and will end 6 weeks postnatally. The last contact for most women will be at approximately 7 weeks post delivery with the receipt of a psychosocial questionnaire. A subset of women consenting to additional follow-up will be contacted for a face-to-face interview after the questionnaire has been returned and a sub-sample of consecutive women will also be asked to complete a second questionnaire at about 16 weeks post delivery to understand patient preferences using a Discrete Choice Experiment (DCE) survey (see Fig. 2).

### Randomisation and blinding

Once consent is obtained and eligibility criteria are met, participants will be randomised to either the treatment group (CTG + STan) or the conventional group (CTG only). Simple randomisation with stratification for parity will be implemented to produce a pre-determined schedule with an allocation ratio of 1:1, with treatment allocations accessed by a telephone-based system provided by NHMRC Clinical Trials Centre at the University of Sydney.

### Description of treatment arms

Continuous electronic fetal monitoring will be conducted by midwives and obstetricians who have been trained in the use of CTG and STan monitoring (holding Australian national Fetal Surveillance Education Program (FSEP) CTG accreditation, as well as in-house, institutional accreditation for competency at STan use and interpretation). Within 6 weeks of birth, all labours and deliveries will be reviewed by a multidisciplinary panel of experienced clinicians to assess adherence to electronic fetal monitoring (CTG and STan) protocols and procedures. Any evidence of protocol and STan guideline violations will be fed back to the relevant providers to optimise protocol adherence.

#### Treatment arm (CTG + STan)

A STan-capable monitor (Neoventa) will be connected to a tocodynometer on a belt applied to the woman’s waist. If her membranes have been ruptured, a FSE will be applied to the occiput of the fetal scalp, and STan monitoring will commence and be interpreted according to the STAN guidelines [27]. If membranes are still intact, they will be artificially ruptured when it is safe and clinically appropriate. A FSE will then be applied, and STan monitoring commenced. If it is not possible or clinically appropriate to rupture the membranes, monitoring via a belt-mounted Doppler monitor will be commenced (as per clinical necessity, using the guidelines of the Royal Australian and New Zealand College of Obstetrics and Gynaecology (RANZCOG) guidelines [28]). Once clinically appropriate and safe, the membranes will be ruptured, a FSE will be applied and STan monitoring will commence.

#### Conventional arm (CTG only)

A CTG machine (Philips or Neoventa) in the delivery room will be activated. A belt with a tocodynometer will be applied to the woman’s waist. External monitoring of the fetal heart rate will commence by a belt-mounted Doppler monitor around her waist or, if clinically indicated, a FSE will be applied to the fetal scalp and monitoring will commence according to the RANZCOG guidelines [28].

### Data collection

Maternal and neonatal data will be collected from retrospective case-note review by the research midwife who is not involved in the primary provision of care. The treatment allocation cannot be concealed because the content of the notes will reveal the care received. An independent review of 20 records will be completed to validate the quality and accuracy of data entry. Outcomes and timepoints of data collection are detailed in Fig. 2. Prospectively collected data will also be obtained in order to construct a CONSORT flow chart [29, 30].

#### Primary outcome

The primary outcome is emergency caesarean section (yes/no).

#### Secondary outcomes

Maternal secondary outcomes include the following: type of labour; indication for induction; type of fetal monitoring received; cardio-monitoring method; number and classification of CTG abnormalities and clinician responses; number and type of ST events and clinician responses; adherence to STAN guidelines; use of and result for scalp pH and/or lactate; use of oxytocin infusion and length of use; complications of labour; length of labour; use of pharmacologic analgesia and non-pharmacologic pain relief; mode of delivery; declared reason if caesarean section and Robson classification; trial of assisted vaginal delivery resulting in caesarean section; postpartum antibiotic use, endometritis and maternal readmission; maternal ICU admission; maternal death; and length of stay.

Neonatal secondary outcomes include the following: intrapartum fetal death; birth weight; gender; intubation or external cardiac massage at delivery; arterial and venous cord gases (pH, lactate, base excess); APGAR score at 1, 5 and 10 min; limb or clavicular fracture; neonatal death; seizure(s); BRAINZ monitoring; presence and grade of neonatal encephalopathy; requirement for cooling; proven infection; antibiotic usage; potential complication from use of fetal scalp electrode; scalp trauma; admission to neonatal intensive care, special care or qualified on ward; length of stay; jaundice requiring phototherapy; respiratory distress; meconium aspiration syndrome; major congenital malformation; other major complications; and neonatal readmission.

#### Additional study outcomes

All participants in the study are offered the opportunity to participate in research observing psychosocial outcomes of the monitoring they received, and a sub-sample of consecutive participants will also be offered the opportunity to participate in research regarding preferences, utilising a DCE survey. In consenting to participate in the randomised controlled trial, participants also agree to being sent questionnaires during the postnatal period.

Women are invited to complete a psychosocial questionnaire which they will receive approximately 7 weeks after delivery, with the exception of women with severe adverse outcomes (e.g. fetal or neonatal death). The questionnaire has been constructed using several measures to examine satisfaction with continuous electronic fetal monitoring, birth satisfaction, general health, postnatal depression and infant feeding practices. These tools have robust psychometric properties and have been successfully applied in the maternity setting in past studies. The tools include the EQ-5D [31], the General Health Questionnaire [32], the Edinburgh Postnatal Depression Scale (EPDS) [33], a measure of infant feeding [34] and the Birth Satisfaction Scale—Revised (BSS-R) [35]. Also included are demographic questions, a purpose-designed scale of monitoring satisfaction, trade-off questions and open-ended questions on positive and negative experiences. A subset of women who return the questionnaire, and indicated they are interested in participating, will be contacted regarding a face-to-face interview in relation to their satisfaction and experience with the continuous electronic fetal monitoring they received.

Women may also be invited to participate in a patient preference survey using a DCE which will examine what trade-offs women are likely to make in order to obtain advantages such as reduced change of emergency caesarean section. A three-stage approach, previously successfully applied [36], will be followed: existing data from a qualitative study [37] conducted during the pilot study will be used to determine the key factors (attributes) influencing preference; a preliminary questionnaire will be developed and piloted, providing data to inform an efficient experimental design for the main survey; and the final DCE questionnaire will be sent to women at approximately 16 weeks postnatal.

Lastly, a cost-effectiveness study will be completed at the end of the randomised controlled trial. Taking a hospital perspective, and utilising cost data generated by hospital systems from all participants in the study, the incremental cost per emergency caesarean section avoided will be calculated.

### Statistical analysis

Analysis of the randomised controlled trial will be performed by the Data, Design, Statistics and Research IT Service of Adelaide Health Technology Assessment (AHTA), School of Public Health, The University of Adelaide. Statistical analysis will follow standard methods for randomised trials and will be performed on an intention-to-treat basis, according to treatment allocation at randomisation. All analyses will follow a pre-specified statistical analysis plan. For dichotomous outcomes including the primary outcome of caesarean section (yes/no), proportions will be compared between treatment groups using the relative risk with 95% CI, obtained from a log binomial regression analysis with adjustment for strata (defined by parity) used in the randomisation. Further log binomial regression analyses (for sensitivity) will examine the effect of adjustment for parity, as well as pre-specified prognostic baseline variables and/or variables not balanced (by chance) at baseline, with results reported as the relative risk with corresponding 95% CIs. Categorical outcomes with more than two categories will be modelled with multinomial or ordinal logistic regression (or an appropriate generalised linear model alternative) with adjustment for parity. Proportions will be compared between treatment groups with relative risks and 95% CIs where possible. Continuous outcomes will be compared using differences between mean values estimated from linear or generalised linear regressions (depending on the nature of the data) adjusted for parity (with 95% CIs). Secondary analyses will explore evidence for heterogeneity of effects between the two parity strata using interaction tests and subgroup analyses. All subgroup comparisons will be pre-specified and performed with appropriate tests of interaction. Although this study will be underpowered to determine equivalence of neonatal outcomes or maternal secondary outcomes, the neonatal data will be able to be incorporated into meta-analyses including neonatal data from previous clinical trials (including the use of individual patient data).

For the psychosocial survey, analysis of the data will be blinded to the specific treatment group, with simple indicators ‘A’ and ‘B’ provided by AHTA.

For the DCE, analysis will begin using multinomial logit (MNL) models and mixed logit models; alternative model specifications (e.g. latent class models) may also be used to explore preference heterogeneity. Models will be evaluated for goodness of fit using the likelihood-ratio χ^2^ statistic for the global test of zero model coefficients, McFadden’s pseudo *R*^2^ and Akaike’s information criterion (AIC). MNL and mixed logit model results will be presented as *β* parameters and odds ratios with 95% confidence intervals. Trade-offs between parameters (e.g. benefit–harm trade-offs) will also be estimated; and will be calculated as the ratio of model parameters.

For the cost-effectiveness study, data collected from clinical feeder systems at the hospital will be calculated on a full absorption basis including labour and postnatal ward, medical and midwifery staff, pathology, pharmacy, operating theatre, medical and surgical supplies, goods and services. Preparation of costing data will conform to Australian Hospital Patient Costing Standards [38]. Direct cost comparisons will use case-mix classifications (Australian Related Diagnosis Groups classification, version 8.0) [39]. The incremental cost per emergency caesarean section avoided will be calculated. Bootstrapping will estimate a distribution around incremental costs and incremental health outcomes, and confidence limits around the incremental cost-effectiveness ratio will be estimated. One-way sensitivity analysis will be conducted around key variables, and a probabilistic sensitivity analysis will be conducted to estimate joint uncertainty in all parameters.

### Data safety and adverse event monitoring and reporting

Adverse events will be monitored for by the clinical investigators and research midwife at ward rounds or perinatal review sessions. The research midwife will also monitor for adverse events as she extracts clinical data from the case notes. Adverse events entered into the data management software will automatically trigger the generation of an email alert to the obstetrician and paediatrician investigators. Additionally, results of the EPDS within the psychosocial questionnaire will be monitored to ascertain offers of referral.

A Data Safety Monitoring Committee (DSMC) has been formed to assess emerging external evidence in relation to STan, and to monitor adverse events, compliance with trial protocol and progress of recruitment. The DSMC will be notified of and meet as soon as possible after the occurrence of any of the following adverse events: maternal death; and fetal or neonatal death that could be reasonably attributed to, or partly caused by, CTG + STan or CTG alone. The DSMC will also be notified of and asked to deliberate on adverse events including unplanned intensive care admissions (maternal or neonatal) and any other adverse event of significant concern to the investigators or clinical staff. The committee will also be convened in the event that the chief investigator (CW) receives a report of an adverse event, due to STan monitoring, from weekly alerts of the published literature. Responses of the DSMC may include cessation of the study or modification of the protocol, which will in turn be re-submitted to the ethics committees.

### Dissemination of results

Results will be disseminated via peer-reviewed publications as well as at institutional and state-based clinical meetings and national and international conferences such as the Perinatal Society of Australia and New Zealand and the Royal College of Obstetrics and Gynaecology. It is expected that the study will also be the subject of journal clubs and Twitter feeds, and we will ensure that the results are translated into relevant clinical guidelines, practice and policy briefs, news releases, educational sessions and one-to-one briefings. Results will also be directly disseminated via the Women’s Healthcare Australasia Clinical Network.

## Discussion

About 20% of Australian babies are delivered via emergency caesarean section [8]. At 35% overall, Australian caesarean section rates continue to remain above the OECD average (34% in 2014) and are among the highest in the world, ranking 24th out of 31 OECD countries (rates ranked lowest to highest) [40]. This will be the first Australian trial to examine the potential for STan technology to reduce this rate. It will also be among the first to consider a more comprehensive examination of STan, taking into account both the impact on broader psychosocial well-being and patient preference, as well as cost-effectiveness. Professional bodies such as the Royal Australian and New Zealand College of Obstetrics and Gynaecology are currently relying on international evidence which our critique demonstrates is not generalisable to Australian practice.

In previous trials, very little attention has been paid to the views of women, midwives and obstetricians. This may partly explain the problems experienced with uptake in some settings [41]. In order to identify associated psychosocial and organisational issues, we have already conducted qualitative research with women throughout the hospital [37] as well as obtaining the views of midwives and obstetricians about the introduction of STan and the accompanying educational programme [42]. In-depth, semi-structured interviews incorporating hypothetical written scenarios of STan and CTG revealed that women’s views about monitoring are multifaceted and likely to be influenced by perceptions related to four key factors: risk in labour; mobility in labour; autonomy and choice in labour; and trust in maternity providers [37]. In contrast, doctors and midwives indicated four important areas for consideration when introducing STan: a philosophy of care; the implementation process (including training and education); the existence of research evidence; and attitudes towards new technology [42].

This research will provide Australian evidence for clinical practice and guideline development which is crucial for policy-makers and consumers to make informed, evidence-based choices about care in labour. Given the ubiquitous application of CTG monitoring, we also anticipate that the trial will initiate and inform professional discussions about monitoring at an international level.

### Trial status

The trial is currently recruiting participants. Current protocol version 28 (13 March 2019). Recruitment commenced in 22 January 2018; expected recruitment completion is July 2020. The primary investigator will notify the trial register, the ethics committees, the DSMC and this journal should any major changes be made to the trial protocol.

## Additional file

Additional file 1: SPIRIT 2013 Checklist: Recommended items to address in a clinical trial protocol and related documents (PDF 116 kb)

## Data Availability

Not applicable.

## Abbreviations

AHTA: Adelaide Health Technology Assessment
CI: Confidence interval
CONSORT: Consolidated Standards of Reporting Trials
CTG: Cardiotocography
DCE: Discrete Choice Experiment
DSMC: Data Safety Monitoring Committee
EPDS: Edinburgh Postnatal Depression Scale
FSEP: Fetal Surveillance Education Program
MNL: Multinomial logit
NHMRC: National Health and Medical Research Council
OECD: Organisation for Economic Co-operation and Development
OR: Odds ratio
RANZCOG: Royal Australasian and New Zealand College of Obstetrics and Gynaecology
RR: Risk ratio
STan: ST analysis
START: STan Australian Randomised controlled Trial
